# The Optimal Activity of Radioactive Iodine for Remnant Ablation in Low/Intermediate Risk Differentiated Thyroid Carcinoma: A Continuous Controversy and Meta-Analysis

**DOI:** 10.7759/cureus.12937

**Published:** 2021-01-27

**Authors:** Hyder Mirghani, Mohammed I Altidlawi, Ibrahim A Altedlawi Albalawi

**Affiliations:** 1 Internal Medicine, University of Tabuk, Tabuk, SAU; 2 Surgery, University of Tabuk, Tabuk, SAU; 3 Surgical Oncology, University of Tabuk, Tabuk, SAU

**Keywords:** radioactive iodine activity, low/high doses, low/intermediate risk, differentiated thyroid carcinoma, remnants ablation

## Abstract

Radioactive iodine (RAI) is widely used for remnant ablation in low/intermediate differentiated thyroid carcinoma (DTC). However, the optimal effective dose that overweighs the benefits over unwanted side effects is a matter of controversy. This meta-analysis aimed to assess low versus high doses of RAI activity for DTC remnant ablation. Two authors independently searched PubMed and Cochrane Library using the keywords low dose radioactive iodine, high dose radioactive iodine, low-risk/intermediate risk, differentiated thyroid carcinoma, and remnant ablation. Two hundred and twenty references were identified when limiting the engine to controlled trials in English and during the period from January 2010 to December 2020. Nine trials (five from Europe and four from Asia) including 3137 patients fulfilled the inclusion and exclusion criteria. The data were then entered in an extraction sheet detailing the trial information including the author's name, year of publication, country, and type of surgery, preparation for RAI, the patients and control number in the low and high-dose groups, follow-up period, and the results. Out of 220 articles retrieved, nine controlled trials were included (follow-up period range, six months to 12 years, 3137 patients, and low risk of bias). The analysis favored the high dose for remnants ablation, odd ratio, 0.73, 95% CI*, *0.50-1.07*; *P-value for the overall effect was 0.10. However, the results were limited due to the significant heterogeneity observed (56%, P-value 0.03). High-dose RAI was better for DTC remnants ablation. Further studies focusing on intermediate-risk DTC and adjusting for preoperative and postoperative factors are recommended.

## Introduction

Drafting a review about the radioactive iodine effective dose is triggered by the controversy surrounding it. The controversy starts from the name: is it a dose? So that it can be viewed like chemotherapy or an activity calculated from the given patients' characters and imaging factors. The arena of the activity against the dose ended in the controversy regarding the protective effects of radioactive iodine (RAI) on solid tumors and the induction of hematological malignancies aside from multiple myeloma [1]. The second reason is the substantial fear stocked among the patients who might be candidates for RAI [2]. Importantly, these gaps were not sufficiently addressed to alleviate the patient's anxiety. Also, the goal of RAI might be hazy in most of the previous studies (remnants ablation, adjuvant, or for metastatic disease) [3]. The previous meta-analyses retrieved add to the above controversy; some showed the superiority of the high dose [4], other studies suggested high doses for incomplete thyroid surgery in certain parts of the World [5-6], the rest showed no differences between high and low doses [7-9]. Besides, no recent updates regarding this important issue. Given the above and the increasing focus on RAI's side effects, including craniofacial and psychosocial effects [10], we conducted this meta-analysis to compare high- and low-dose RAI for low/intermediate differentiated thyroid carcinoma remnants ablation. The current review thought to address the type of surgery, preparation for RAI, and the postoperative assessment methods ignored by previous studies.

## Materials and methods

Inclusion criteria according to Population, Intervention, Comparison, Outcomes, and Study (PICOS)

Studies were included if they are randomized control trials in the English language and comparing low-dose and high-dose radioactive iodine. The studies should have been carried out among adults with low/intermediate-risk differentiated thyroid carcinoma (DTC). Methodologies other than controlled trials, not specifying the dose, and carried on other thyroid disorders (thyrotoxicosis, non-differentiated thyroid carcinoma, and high-risk DTC) were excluded. Studies were approached if they stated at least two postoperative follow-up measures to assess the outcomes.

Patients

Adults with the diagnosis of DTC and followed for at least six months, children, and pregnant women who might not be candidates for radioactive iodine were not included. We included patients with total or near-total thyroidectomies with possible lymph node dissection. Thyroid hormone withdrawal with and without recombinant thyroid-stimulating hormone (TSH) was accepted. However, we did not control for TSH levels before the administration of RAI. The patients must be followed by at least two postoperative investigations to assess the outcomes (thyroid ultrasonography, neck scan, whole-body scan, stimulated thyroglobulin, and thyroglobulin antibodies). In the present meta-analysis, both 1100 and 1850 MBq were considered low doses while 3700 was considered a high dose [11]. An example is Kukulska et al. [10], whose study was in two stages: first comparing 30 mCi and 60 mCi and in the second, the authors assessed 60 vs 100 mCi and showed no differences. Thus, we combined 30 mCi and 60 mCi as low doses.

Literature search

**Figure 1:**
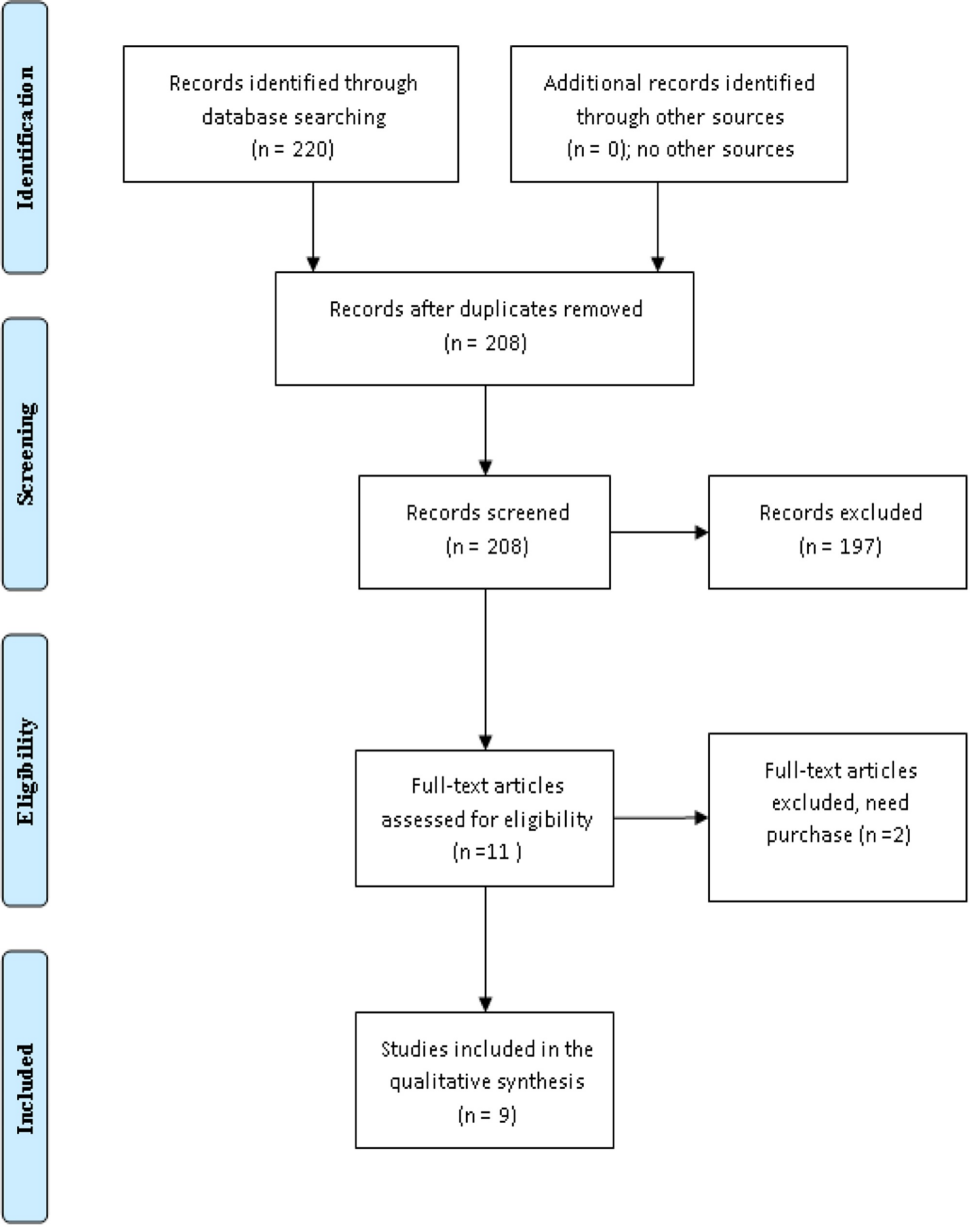
Trials included in a comparison between low and high doses of radioactive iodine for differentiated thyroid carcinoma remnants ablation

The search engine was set to controlled trials in English in PubMed and the Cochrane Library. The keywords used were low dose radioactive iodine, high dose radioactive iodine, low-risk differentiated thyroid carcinoma, intermediate-risk differentiated thyroid carcinoma, and remnant ablation. The systematic literature search was limited to the period from January 2010 to December 2020. We identified 220 references (204 in PubMed and 16 in the Cochrane library), of the 11 studies, which fulfilled the inclusion and exclusion criteria, and two were excluded (purchase is a need). Thus, nine full texts were included in the final meta-analysis. The results were exported to a data analysis sheet including the author's names, year of publication, country, type of surgery, preparation for RAI, the patients and controls numbers in the low and high doses groups, the follow-up period, and the results. A modified Cochrane risk of bias tool was used to assess the quality of the included trials (Figure 1, Table 1) [12].

**Table 1:** The included trials' patient characteristics

Author	Type of Surgery	Preparation for RAI	Postoperative assessment
Kukulska et al. 2010 [10]	TT & possible lymph node dissection	Thyroid hormone withdrawal	Neck scan and the stimulated thyroglobulin
Caglar et al.2011 [13]	Total thyroidectomy	Thyroid hormone withdrawal	Neck scan, ultrasound, and the stimulated thyroglobulin
Fallahi et al. 2011 [14]	TT	Thyroid hormone withdrawal	Neck scan, ultrasound, and the stimulated thyroglobulin
Ma et al. 2017 [15]	Total or near-total thyroidectomy &possible lymph node dissection	Thyroid hormone withdrawal	Neck scan, whole-body scanning, and serum thyroglobulin.
Mallick et al.2012 [16]	TT & possible lymph node dissection	Contrasted both withdrawal and recombinant TSH	Neck scan, whole-body scan, stimulated thyroglobulin and thyroglobulin antibodies
Qu et al. 2017 [17]	TT & possible lymph node dissection	Thyroid hormone withdrawal	Neck scan and thyroglobulin level
Schlumberger et al. 2012 [18]	TT & possible lymph node dissection	Contrasted both withdrawal and recombinant TSH	Neck scan, whole-body scan, stimulated thyroglobulin and thyroglobulin antibodies
Schlumberger et al. 2018 [19]	TT & possible lymph node dissection	Contrasted both withdrawal and recombinant TSH	Neck scan, whole-body scan, stimulated thyroglobulin and thyroglobulin antibodies
Dehbi et al. 2019 [20]	One or two-stage TT & possible lymph nodes dissection	Contrasted both withdrawal and recombinant TSH	Neck scan, whole-body scan, stimulated thyroglobulin and thyroglobulin antibodies

Outcomes

The studies were included if they assessed remnant ablation among patients with low/intermediate-risk DTC. Outcomes such as adjuvant therapy and RAI used for metastatic thyroid carcinoma were not eligible.

Data analysis

The dichotomous data were manually entered in the Revman system (version 5.4, The Nordic Cochrane Centre, The Cochrane Collaboration, Copenhagen) for reviews with a 95% confidence interval. The mean difference and heterogeneity estimation were conducted. The fixed or random effects were applied depending on the level of heterogeneity observed (> 50% was considered as high). P <0.05 was considered significant. A funnel plot assessed sensitivity.

## Results

In the present data, six out of the nine included trials [10,13-17] were on the side of the higher radioactive iodine for low/intermediate differentiated thyroid carcinoma remnants ablation while three [18-20] were neutral or favored the low dose. The trials were from Europe (five) and Asia (four) and included 3137 patients followed for six months to 12 years. The included studies were assessed by a modified Cochrane system and showed low risk regarding selection, attrition, and reporting while one study (Dehbi et al., 2019) showed a high performance bias. The performance of another four studies was unclear (Kukulska et al., Schlumberger et al., Mallick et al., and Qu et al.) (Table 1). Three of the included studies assessed intermediate-risk differentiated thyroid carcinoma while five were on low-intermediate risk tumors. Of the eight studies included, seven showed that low and high-dose RAI activity was equally effective and one showed the superiority of the high dose. In the present meta-analysis, there were 758 and 775 total events in the interventional and control groups, respectively.

**Figure 2:**
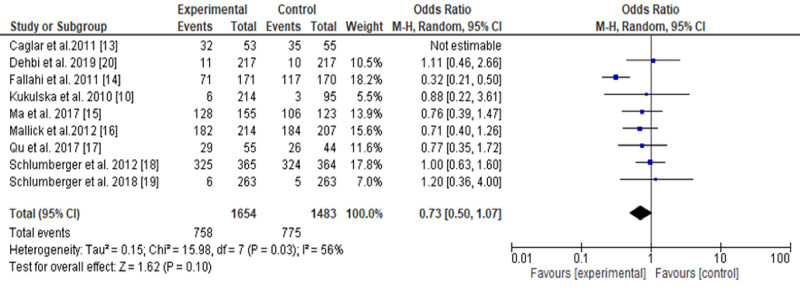
A comparison between low-dose and high-dose radioactive iodine for low/intermediate-risk differentiated thyroid carcinoma

Due to the significant heterogeneity observed (56%, P-value 0.03), the random effect was chosen. The chi-square value was 15.98, Tau2 0.15, and df. 7. The P-value for the overall effect was 0.10, odds ratio 0.73, 95% CI, 0.50-1.07. The results imply that the high dose is more efficacious. The funnel plot showed marked lateralization (Tables 1-3, Figure 2).

**Table 2:** The included trials risk of bias as assessed by the modified revised Cochrane risk of bias tool for randomized trials

Trial	Selection	Performance	Attrition	Reporting	Other
Kukulska et al. 2010	Low	Unclear	Low	Low	Low
Schlumberger et al. 2018	Low	Unclear	Low	Low	Low
Dehbi et al. 2019	Low	High	Low	Low	Low
Caglar et al. 2011	Low	Low	Low	Low	Low
Fallahi et al. 2011	Low	Low	Low	Low	Low
Mallick et al. 2012	Low	Unclear	Low	Low	Low
Schlumberger et al. 2012	Low	Low	Low	Low	Low
Ma et al. 2017	Low	Low	Low	Low	Low
Qu et al. 2017	Low	Unclear	Low	Low	Low

**Table 3:** Controlled trials assessing low versus high-dose radioactive iodine in differentiated thyroid carcinoma remnants ablation

Author	Country	Follow-up/years	Low dose	High dose	Significance
Kukulska et al. 2010 [10]	Poland (low-risk)	2-12	6/214	3/95	Equally effective
Caglar et al. 2011 [13]	Iran (low-risk)	12	32/53	35/55	Equally effective
Fallahi et al. 2011 [14]	Turkey (low/intermediate)	6-12	71/171	117/170	High dose better
Ma et al. 2017 [15]	China (low/intermediate)	2	128/155	106/123	Equally effective
Mallick et al. 2012 [16]	UK (low/intermediate)	6-9	182/214	184/207	Equally effective
Qu et al. 2017 [17]	China (low/intermediate)	2.25	29/55	26/44	Equally effective
Schlumberger et al. 2012 [18]	France (low-risk)	6-10 months	325/365	324/364	No difference
Schlumberger et al. 2018 [19]	France (low-risk)	0.5-9.2	6/263	5/263	No differences
Dehbi et al. 2019 [20]	UK (low/intermediate)	6.5	11/217	10/217	The same recurrence

**Figure 3:**
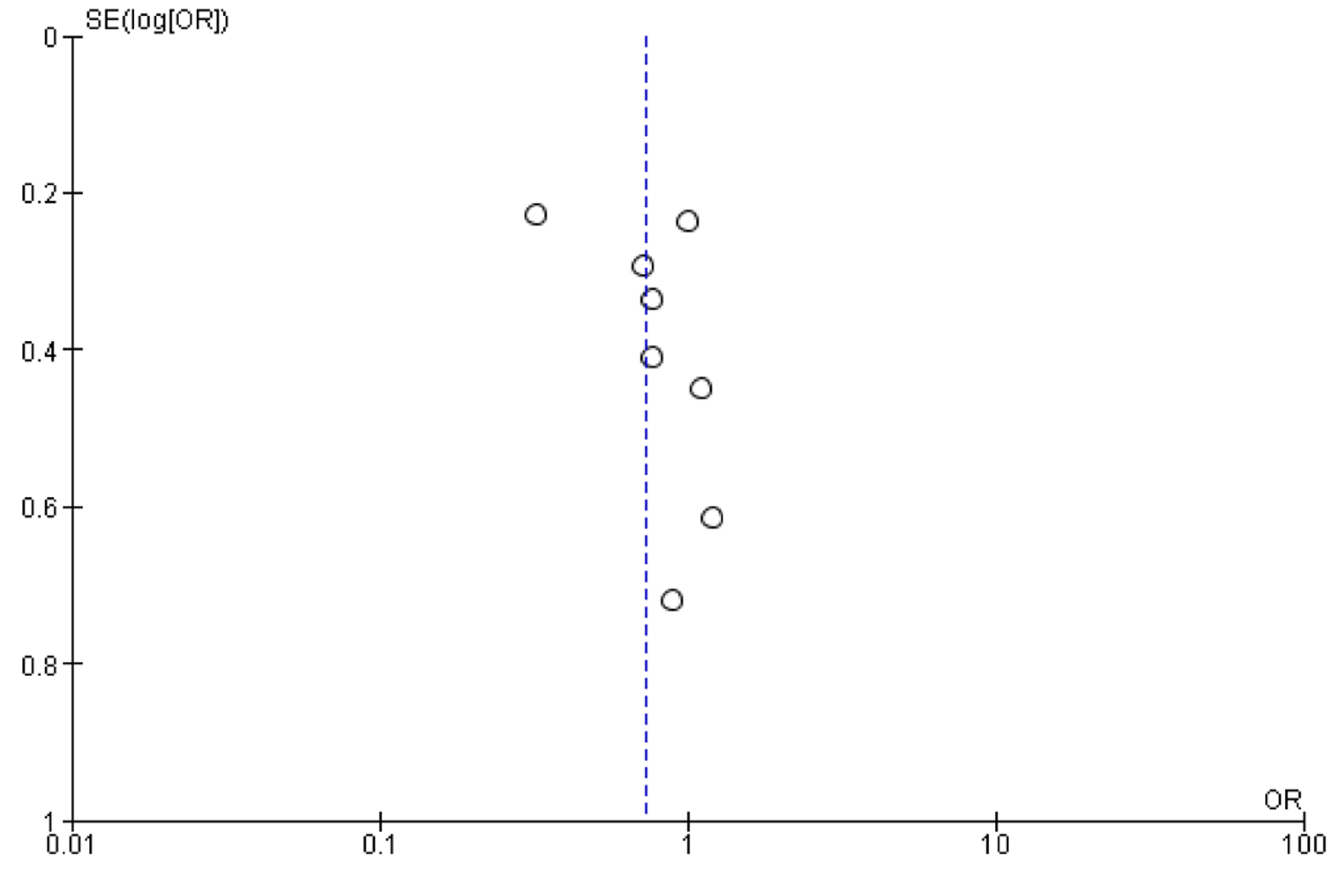
A comparison between low-dose and high-dose radioactive iodine for low/intermediate-risk differentiated thyroid carcinoma

## Discussion

The current finding of the superiority of the high dose radioactive iodine for low/intermediate-risk differentiated thyroid carcinoma remnants ablation might enrich and upgrade the available evidence. The management of DTC with radioactive iodine requires further clarification; the debate is living continuously due to the development in technology [21]. The current finding is in line with Song et al. [4] and Shengguang et al. [6] who compared 1.1 GBq and 3.7 GBq; also, the later study restricted its observations to Europe and following incomplete thyroid surgery. A strength of this meta-analysis is that we defined both 1100 and 1850 MBq as a low dose [11]. Besides, we included both thyroxine withdrawal and recombinant TSH for RAI preparation due to the comparable efficacy [22-23]. A further strength of our data is specifying at least two methods for postoperative assessment due to the limitations and less reliability. Neck ultrasound is operator dependent and less sensitive in the central compartment, a thyroglobulin assay lacks a precise value and is prone to interference by thyroglobulin antibodies, while neck scintigraphy uses different isotopes with varying sensitivity and specificity [3,24]. The current findings were different from previous meta-analyses that recommended low-dose activity [7- 8]. Importantly, Ma et al. included only three trials under recombinant human thyrotropin (rhTSH) stimulation [7]. The pooling of both low and intermediate-risk DTC might explain the discrepancy observed between different meta-analyses. Furthermore, DTC is not uniform (a follicular variant of papillary thyroid carcinoma versus the classical variant and Hurthle cell carcinoma may behave differently). Thus, pooling these sub-groups may affect the results. An important issue that may affect RAI outcomes and inform physicians is the thyroglobulin/TSH ratio. Currently, a TSH of ≥30 mIU/L is suggested for thyroglobulin estimation, however, a great doubt was thrown and this threshold has been questioned. The thyroglobulin/TSH ratio from 0.126 to 0.034 shown by previous studies is wide and needs further adjustments due to its importance to RAI outcomes. Besides, further studies focusing on different DTC sub-types and concentrating on intermediate-risk patients are needed.

## Conclusions

High-dose RAI is recommended for low/intermediate DTC remnant ablation. However, the results were limited by the high heterogeneity observed. Further studies assessing the different DTCs and controlling for TSH levels and postoperative assessment methods are recommended.
